# Comparative Cardiovascular Pharmacovigilance of Semaglutide and Tirzepatide: A Food and Drug Administration Adverse Event Reporting System (FAERS) Database Analysis (2022-2026)

**DOI:** 10.7759/cureus.113371

**Published:** 2026-07-25

**Authors:** Abhinandan Singla, Gurnoor Kaur, Gurpreet Singh, Shivansh Luthra, Chirag Lodha, Omar Alneser, Mohammed S Alo, Syed S Ali, Muhammad Zulqarnain

**Affiliations:** 1 Internal Medicine, Mercy St. Vincent Medical Center, Toledo, USA; 2 Medicine, Government Medical College, Amritsar, IND; 3 Internal Medicine, University of South Florida Morsani College of Medicine, Tampa, USA; 4 Internal Medicine, Henry Ford Macomb Hospital, Detroit, USA; 5 Cardiology, Mercy St. Vincent Medical Center, Toledo, USA; 6 Interventional Cardiology, Mercy St. Vincent Medical Center, Toledo, USA

**Keywords:** arrhythmia, cardiovascular safety, disproportionality analysis, faers, gip receptor agonist, glp-1 receptor agonist, pharmacovigilance, reporting odds ratio, semaglutide, tirzepatide

## Abstract

Background: Semaglutide and tirzepatide are primary treatments for cardiometabolic disease, yet their comparative real-world cardiovascular safety requires further characterization.

Objective: This study aimed to conduct a pharmacovigilance disproportionality analysis comparing cardiovascular adverse event (AE) signals between semaglutide and tirzepatide utilizing Food and Drug Administration Adverse Event Reporting System (FAERS) data from January 2022 to March 2026.

Methods: Cardiac AEs for both medications were extracted from the FAERS database. Disproportionality was evaluated using reporting odds ratios (RORs) and proportional reporting ratios (PRRs), utilizing each drug as the other's comparator. Positive signals required an ROR of >1.0 and a lower 95% CI bound of >1.0.

Results: Analysis included 56,799 semaglutide and 135,992 tirzepatide reports. Overall, cardiac AE reporting was disproportionately favorable to semaglutide (ROR: 2.85; 95% CI: 2.68-3.04). Semaglutide exhibited significant signals in eight of 12 cardiac categories, notably congestive heart failure (ROR: 8.09), cardiac failure (ROR: 3.69), cardiac arrest (ROR: 3.42), and atrial fibrillation (ROR: 3.37). Fatal outcomes also favored semaglutide (ROR: 3.14). Tirzepatide generated higher raw counts for palpitations and tachycardia, though volume adjustment maintained semaglutide's disproportionality.

Conclusions: Semaglutide demonstrates robust, disproportionate cardiovascular AE signals relative to tirzepatide, particularly for structural events. These findings likely reflect underlying demographic and indication-based risk differences rather than direct toxicity. Prospective, head-to-head cardiovascular trials remain essential.

## Introduction

The convergence of type 2 diabetes mellitus (T2DM) and obesity has catalyzed the development of novel pharmacological agents targeting the incretin axis [1]. Glucagon-like peptide-1 (GLP-1) receptor agonists, indicated primarily for T2DM and chronic weight management, have demonstrated efficacy across glycemic, weight, and cardiovascular endpoints [2,3], culminating in semaglutide's demonstrated 20% reduction in major adverse cardiovascular events (MACE) in the SELECT trial among individuals with obesity and established cardiovascular disease but without T2DM [4,5]. Tirzepatide, the first approved dual GLP-1/glucose-dependent insulinotropic polypeptide (GIP) receptor agonist [6], demonstrated superior weight reduction versus semaglutide in the SURPASS-2 and SURMOUNT-5 trials, with cardiovascular outcomes data from SURPASS-CVOT awaited [7,8].

Despite robust randomized controlled trial (RCT) evidence supporting cardiometabolic benefits of both agents, RCTs are inherently limited in their capacity to characterize real-world safety. Strict eligibility criteria exclude patients with complex comorbidities, high polypharmacy burden, or atypical presentations. Trial durations may be insufficient to detect delayed safety signals, and statistical power for uncommon adverse events (AEs), particularly arrhythmias, myocarditis, and sudden cardiac death, may be inadequate [9]. The Food and Drug Administration Adverse Event Reporting System (FAERS) is a cornerstone of post-marketing pharmacovigilance, aggregating spontaneous AE reports from healthcare professionals, consumers, and manufacturers globally [10]. Disproportionality analysis, the statistical backbone of FAERS signal detection, identifies AEs reported at higher-than-expected rates for a drug relative to a comparator, without requiring exposure denominators. The proportional reporting ratio (PRR) and reporting odds ratio (ROR) are the most widely utilized metrics in regulatory pharmacovigilance [11].

Prior FAERS analyses have characterized individual GLP-1 receptor agonist signals for thyroid neoplasms, pancreatitis, and gastrointestinal AEs [9,12]. However, a systematic head-to-head disproportionality comparison of cardiovascular AEs between semaglutide and tirzepatide has not been published. Given the rapid expansion in prescribing volumes for both agents, driven by weight management indications alongside T2DM, and the clinical importance of cardiovascular monitoring in their target populations, such an analysis is both timely and necessary.

This study presents a structured comparative disproportionality analysis of cardiovascular AEs reported to FAERS for semaglutide and tirzepatide from January 2022 through December 2026, spanning tirzepatide's complete post-approval surveillance window to date.

## Materials and methods

Data source and case retrieval

De-identified data were extracted from the FAERS public dashboard and downloadable quarterly American Standard Code for Information Interchange (ASCII) files for the period January 1, 2022, through March 31, 2026 [13]. Reports implicating semaglutide (Ozempic®, Rybelsus®, Wegovy®; Novo Nordisk, Bagsværd, Denmark) or tirzepatide (Mounjaro®, Zepbound®; Eli Lilly, Indianapolis, Indiana, United States) as the primary or secondary suspect drug were identified using drug name and active substance fields. Duplicate reports were identified by case number and date of event and deduplicated by retaining the most recent version. Reports with missing or unresolvable drug identification, or event dates outside the study window, were excluded.

AE classification

Cardiac AEs were identified using the Medical Dictionary for Regulatory Activities (MedDRA) Version 26.0 preferred terms [14]. Events were classified into three clinical categories: (1) arrhythmia-type events: palpitations, atrial fibrillation, tachycardia, bradycardia, ventricular fibrillation, torsade de pointes, and arrhythmia not otherwise specified (NOS); (2) structural/mechanical events: cardiac arrest, cardiac failure, and congestive heart failure; and (3) inflammatory cardiac events: pericarditis and myocarditis. Outcome field coding was used to identify reports with a fatal outcome.

Disproportionality analysis

Disproportionality was assessed using the PRR and ROR with 95% CI, employing a two-drug comparator framework in which each agent served as the reference for the other. For each AE of interest, a 2×2 contingency table (Table 1) was constructed.

**Table 1:** 2×2 contingency table framework for disproportionality analysis For this contingency table, the variables are defined as follows: (a) number of target AE reports for semaglutide, (b) number of all other AE reports for semaglutide, (c) number of target AE reports for tirzepatide, and (d) number of all other AE reports for tirzepatide. AE: adverse event

	Target AE	All other AEs
Semaglutide	a	b
Tirzepatide	c	d

$\mathrm{PRR}=(\mathrm{a/[a+b]})/(\mathrm{c/[c+d]})$. $\mathrm{ROR}=(\mathrm{a/b})/(\mathrm{c/d})=(\mathrm{a&times;d})/(\mathrm{b&times;c})$. The 95% CI for ROR was calculated as follows: $\mathrm{exp(ln[ROR]&plusmn;1.96&times;&radic;[1/a+1/b+1/c+1/d])}$. A pharmacovigilance signal was defined as ROR >1.0 with the lower bound of the 95% CI >1.0, and a PRR ≥2 with a chi-square ≥4 and at least three cases, consistent with the criteria of Evans et al. [11]. All calculations were performed using Python 3.12 (Python Software Foundation, Wilmington, Delaware, United States) with the SciPy (SciPy Developers) and NumPy (NumPy Developers) libraries. Chi-squared tests with Yates correction were applied to 2×2 tables; p<0.05 was considered statistically significant.

Demographic analysis

Reporter-submitted sex (male, female, not specified) and age category (two months to two years; 12-17 years; 18-64 years; 65-85 years; >85 years) were extracted for available reports. Distributions were compared descriptively between drug groups.

Ethical and regulatory considerations

While the FAERS database contains de-identified data and is publicly available and the study is exempt from formal institutional review board oversight under 45 CFR §46.101(b), we confirm that this retrospective database analysis inherently involved human data. This analysis adhered to the International Society for Pharmacoepidemiology (ISPE) Good Pharmacoepidemiology Practices guidelines and the Consolidated Standards of Reporting Trials (CONSORT) extension for pharmacovigilance studies [15]. No patient consent was required.

## Results

Reporting volume and overall cardiac signal

Over the study period, 56,799 total AE reports were identified for semaglutide and 135,992 for tirzepatide, a 2.39-fold difference in raw reporting volume. Total cardiac AEs numbered 2,183 (3.84%) for semaglutide and 1,879 (1.38%) for tirzepatide. Disproportionality analysis of the overall cardiac AE category yielded a strongly positive signal for semaglutide: ROR: 2.85 (95% CI: 2.68-3.04) and PRR: 2.78. The death signal was similarly semaglutide-disproportionate: ROR: 3.14 (95% CI: 2.23-4.42) and PRR: 3.14.

Disproportionality by cardiac AE subtype

Table 2 presents the full disproportionality analysis by MedDRA preferred term. Statistically significant signals were detected for semaglutide in eight of 12 AE categories. The four non-significant categories (pericarditis, ventricular fibrillation, torsade de pointes, myocarditis) had low absolute counts in both groups, resulting in wide confidence intervals. No statistically significant signal favoring tirzepatide was detected for any cardiac AE category.

**Table 2:** Cardiac AE disproportionality analysis: semaglutide vs. tirzepatide (FAERS 2022-2026) Data are represented as raw counts (n) and percentages (%). Percentages represent the proportion of the specific AE out of total cardiac AEs. Contingency variables used for analysis: (a) target AE reports for semaglutide; (b) all other AE reports for semaglutide; (c) target AE reports for tirzepatide; and (d) all other AE reports for tirzepatide. Disproportionality was analyzed using chi-squared tests with Yates correction, and statistical significance was defined as p<0.05. Total semaglutide reports: n=56,799; total tirzepatide reports: n=135,992. MedDRA PT: Medical Dictionary for Regulatory Activities Preferred Term; PRR: proportional reporting ratio; ROR: reporting odds ratio; 95% CI: 95% confidence interval; NS: not statistically significant (lower 95% CI bound ≤1.0); NOS: not otherwise specified; AE: adverse event; Sema: semaglutide; Tirz: tirzepatide; FAERS: Food and Drug Administration Adverse Event Reporting System

AE (MedDRA PT)	Sema n (%)	Tirz n (%)	PRR	ROR	95% CI	Signal	Category
Arrhythmia-type events							
Palpitations	415 (19%)	578 (30.8%)	1.72	1.72	1.52-1.96	Sema > Tirz	Arrhythmia
Atrial fibrillation	285 (13.1%)	203 (10.8%)	3.36	3.37	2.82-4.04	Sema > Tirz	Arrhythmia
Tachycardia	183 (8.4%)	282 (15%)	1.55	1.56	1.29-1.87	Sema > Tirz	Arrhythmia
Arrhythmia (NOS)	76 (3.5%)	66 (3.5%)	2.76	2.76	1.98-3.84	Sema > Tirz	Arrhythmia
Bradycardia	26 (1.2%)	18 (1%)	3.46	3.46	1.90-6.31	Sema > Tirz	Arrhythmia
Ventricular fibrillation	8 (0.4%)	11 (0.6%)	1.74	1.74	0.70-4.33	NS	Arrhythmia
Torsade de pointes	1 (0.05%)	1 (0.05%)	2.39	2.39	0.15-38.28	NS	Arrhythmia
Structural/mechanical events							
Cardiac arrest	107 (4.9%)	75 (4%)	3.42	3.42	2.55-4.60	Sema > Tirz	Structural
Cardiac failure	94 (4.3%)	61 (3.2%)	3.69	3.69	2.68-5.10	Sema > Tirz	Structural
Congestive heart failure	81 (3.7%)	24 (1.3%)	8.08	8.09	5.13-12.76	Sema > Tirz	Structural
Inflammatory events							
Pericarditis	9 (0.4%)	13 (0.7%)	1.66	1.66	0.71-3.88	NS	Inflammatory
Myocarditis	1 (0.05%)	6 (0.3%)	0.40	0.40	0.05-3.31	NS	Inflammatory
Overall cardiac AEs	2,183	1,879	2.78	2.85	2.68-3.04	Sema > Tirz	-
Death (fatal outcome)	76	58	3.14	3.14	2.23-4.42	Sema > Tirz	-

**Figure 1:**
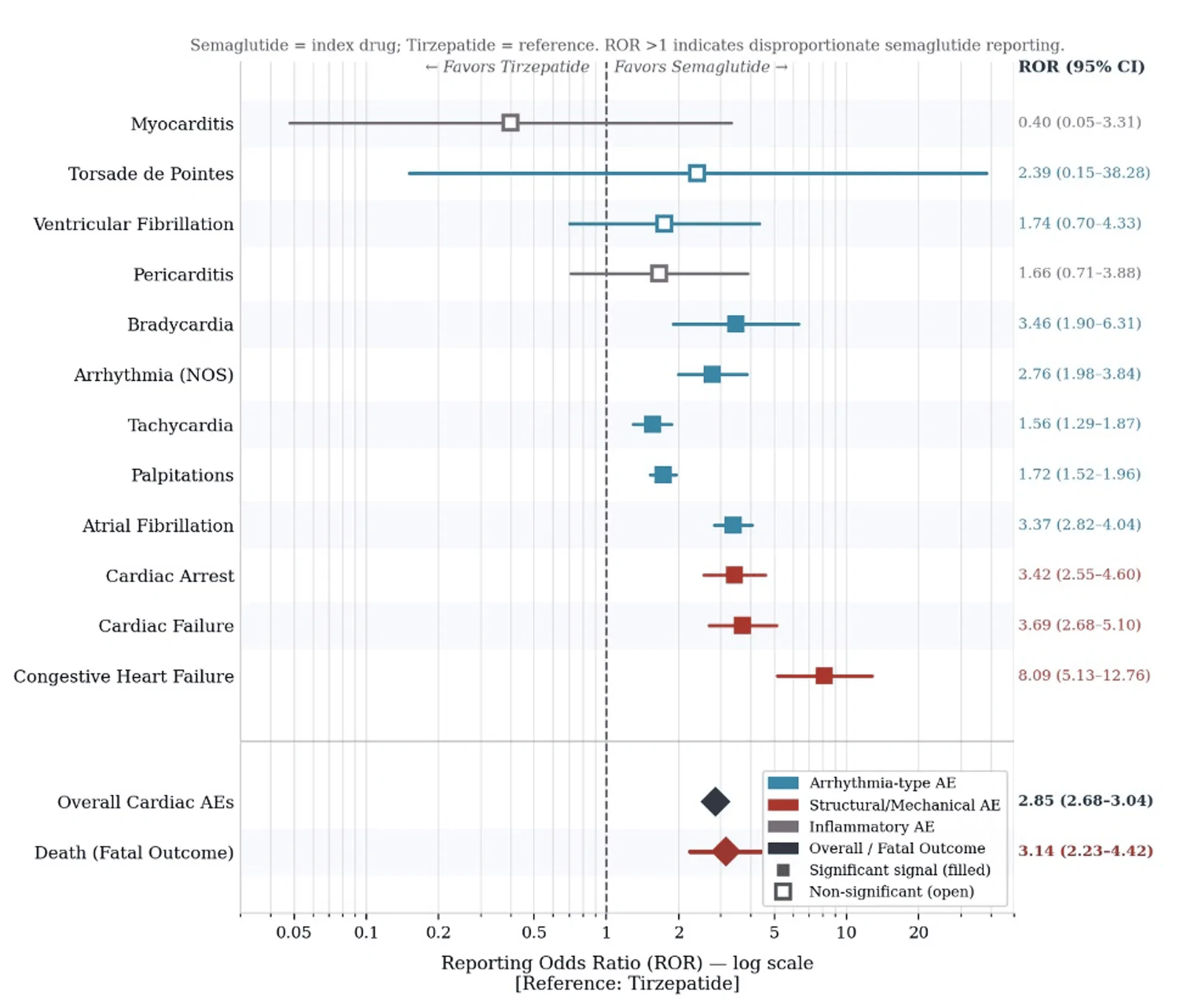
Forest plot of ROR for cardiovascular AEs comparing semaglutide and tirzepatide Data are represented as ROR with 95% CI. Disproportionality was analyzed using chi-squared tests with Yates correction. Statistical significance was defined as p<0.05. ROR: reporting odds ratio; 95% CI: 95% confidence interval; NOS: not otherwise specified; AE: adverse event; FAERS: Food and Drug Administration Adverse Event Reporting System

Figure 1 shows the forest plot of ROR for cardiovascular AE comparing semaglutide and tirzepatide.

Structural cardiac events: congestive heart failure, cardiac failure, and cardiac arrest

The most striking disproportionality was observed for congestive heart failure (ROR: 8.09; 95% CI: 5.13-12.76), with 81 (3.7%) semaglutide reports versus only 24 (1.3%) for tirzepatide after adjusting for denominator volumes. Cardiac failure (ROR: 3.69; 95% CI: 2.68-5.10) and cardiac arrest (ROR: 3.42; 95% CI: 2.55-4.60) also showed highly significant semaglutide-disproportionate signals. These findings are clinically important and require nuanced interpretation in the context of known prescribing patterns (see Discussion).

Arrhythmia signals

Atrial fibrillation demonstrated one of the strongest disproportionality signals in the arrhythmia category (ROR: 3.37; 95% CI: 2.82-4.04), with 285 (13.1%) semaglutide versus 203 (10.8%) tirzepatide reports. This is particularly notable given that tirzepatide had a 2.4-fold higher total reporting volume, meaning the absolute excess of atrial fibrillation reports with semaglutide represents a large proportional difference after volume adjustment.

A counterintuitive finding emerges for palpitations and tachycardia: despite higher raw counts for tirzepatide (578 (30.8%) and 282 (15%), respectively, versus semaglutide's 415 (19%) and 183 (8.4%)), after total reporting volume adjustment, semaglutide shows statistically significant disproportionality for both (palpitations: ROR: 1.72 and 95%: CI 1.52-1.96; tachycardia: ROR: 1.56 and 95% CI: 1.29-1.87). This indicates that while tirzepatide generates more raw reports of palpitation/tachycardia, the excess is proportional to, or slightly less than, tirzepatide's higher overall reporting volume. Both signals remain elevated for semaglutide after adjustment.

Inflammatory cardiac events

No statistically significant disproportionality signal was detected for pericarditis (ROR: 1.66; 95% CI: 0.71-3.88) or myocarditis (ROR 0.:40; 95% CI: 0.05-3.31). The wide confidence intervals for both reflect small absolute case counts (nine (0.4%) and one (0.05%) for semaglutide; 13 (0.7%) and six (0.3%) for tirzepatide), limiting interpretability. The point estimate for myocarditis numerically favors tirzepatide, but this should be considered hypothesis-generating only.

Demographics

As shown in Table 3, females constituted approximately two-thirds of reporters for both agents.

**Table 3:** Reporter demographics: sex distribution

	Male n (%)	Female n (%)	Not specified n (%)	Percentage of females
Tirzepatide	268 (21.2%)	831 (65.6%)	167 (13.2%)	75.6%
Semaglutide	366 (30.6%)	749 (62.6%)	81 (6.8%)	67.2%

A clinically important age distribution difference was identified: the 65-85-year cohort represented 39.2% (n=306) of semaglutide cardiac AE reporters versus 29.8% (n=258) for tirzepatide. This 9.4 percentage-point difference in older adult representation is consistent with semaglutide's established cardiovascular outcomes indication and its preferential use in elderly, higher cardiovascular risk populations, a demographic factor that substantially confounds direct comparison of AE rates between these agents (Table 4).

**Table 4:** Reporter demographics: age distribution Age data from a subset of reports with demographic information available (semaglutide: n=781; tirzepatide: n=867).

Age category	Semaglutide n (%)	Tirzepatide n (%)
2 months-2 years	0 (0%)	1 (0.1%)
12-17 years	2 (0.3%)	1 (0.1%)
18-64 years	470 (60.2%)	605 (69.8%)
65-85 years	306 (39.2%)	258 (29.8%)
>85 years	3 (0.4%)	2 (0.2%)

## Discussion

This is, to our knowledge, the first published head-to-head disproportionality analysis of cardiovascular AEs for semaglutide versus tirzepatide using FAERS data across their concurrent post-marketing periods. Our principal finding, a broadly semaglutide-disproportionate cardiovascular AE signal across eight of 12 MedDRA cardiac preferred terms, merits careful mechanistic, epidemiological, and clinical interpretation before any inference of comparative drug toxicity is drawn.

Confounding by indication and population risk

The most parsimonious explanation for the observed semaglutide-disproportionate signals is confounding by cardiovascular indication. Semaglutide carries an explicit FDA-approved indication for reducing cardiovascular risk in adults with T2DM and established cardiovascular disease (Ozempic® label [16]) and for reducing MACE in adults with obesity and established cardiovascular disease (Wegovy® label [17], SELECT trial [4]). Tirzepatide is approved for T2DM management and chronic weight management, but not for cardiovascular outcomes, at the time of this analysis. Consequently, semaglutide is preferentially prescribed to patients who, by definition, have higher baseline cardiovascular risk, and these patients are more likely to experience cardiac AEs regardless of drug exposure [18]. The demographic data support this interpretation: 39.2% (n=306) of semaglutide cardiac AE reporters were aged 65-85 years, compared with 29.8% (n=258) for tirzepatide. This older-cohort disproportionality likely underestimates the true underlying cardiovascular risk differential, as age is an imperfect proxy for comorbidity burden, prior myocardial infarction, ejection fraction, and polypharmacy.

Structural cardiac signals: congestive heart failure and cardiac failure

The congestive heart failure signal (ROR: 8.09; 95% CI: 5.13-12.76) is the most numerically prominent finding in this analysis. Semaglutide's post-marketing use encompasses patients with heart failure with preserved ejection fraction (HFpEF), where the SELECT trial population included many such individuals, and the STEP-HFpEF trial demonstrated symptom and exercise capacity benefits of semaglutide in this subgroup [19]. The high congestive heart failure signal likely reflects a combination of real-world prescribing to patients with existing heart failure and disproportionate reporting of cardiac decompensation events in this population. This interpretation is consistent with the known reporting behavior dynamics in FAERS, where drug use in populations with severe underlying disease generates disproportionate serious event reports.

There is no mechanistic basis to suggest semaglutide causes de novo congestive heart failure; GLP-1 receptor agonists are not known to have negative inotropic effects, and observational data consistently show neutral-to-favorable effects on cardiac structure and function. The signal should therefore be interpreted as a marker of patient population characteristics rather than direct drug toxicity.

Atrial fibrillation

The atrial fibrillation signal for semaglutide (ROR: 3.37; 95% CI: 2.82-4.04) is consistent with prior FAERS analyses of the GLP-1 class. Storgaard et al. and subsequent analyses have noted that GLP-1 receptor agonist use is associated with atrial fibrillation reporting in pharmacovigilance databases, though this may partly reflect the high background atrial fibrillation prevalence in the obese, diabetic, and elderly patients receiving these drugs [9]. The GLP-1 receptor is expressed in atrial cardiomyocytes and sinoatrial nodal tissue, and experimental data suggest potential electrophysiological effects, though the clinical significance of these effects in humans is not established [20].

The absence of a tirzepatide-disproportionate atrial fibrillation signal, despite tirzepatide's higher overall reporting volume, is noteworthy. Whether GIP co-agonism confers atrial protection, or whether this reflects population differences in baseline atrial fibrillation prevalence, cannot be determined from FAERS data. Prospective data from SURPASS-CVOT will be important in resolving this question.

Palpitations and tachycardia: interpreting the paradox

The apparent paradox of higher raw counts of palpitations and tachycardia with tirzepatide, alongside semaglutide-disproportionate ROR signals, warrants explanation. After adjusting for the 2.39-fold difference in total reporting volume, semaglutide's cardiac event rate remains proportionally higher across all AE categories, including palpitations and tachycardia. However, in absolute terms, tirzepatide's palpitation and tachycardia burden is substantial, and the magnitude of proportional excess for semaglutide in these categories (ROR: 1.56-1.72) is the lowest in this analysis. This suggests that while semaglutide retains a statistical signal after adjustment, tirzepatide does generate a clinically meaningful palpitation/tachycardia burden that is broadly attributable to GIP receptor-mediated cardiac chronotropy, consistent with the 1-2 bpm heart rate increase observed with tirzepatide versus semaglutide in head-to-head trials [21].

Fatality signal

The death signal (ROR: 3.14; 95% CI: 2.23-4.42) requires careful contextualization. FAERS fatal outcome coding does not imply causation; it indicates that death was reported as an outcome in the same report as the suspect drug. Given that semaglutide is used in a higher cardiovascular risk and older population with explicit cardiovascular indications, deaths in this cohort are more likely to represent the natural history of severe cardiovascular disease in a vulnerable population rather than drug-attributable mortality. This interpretation is directly supported by the SELECT trial, which demonstrated a 20% reduction in cardiovascular mortality with semaglutide versus placebo in the same high-risk population.

Strengths and limitations

Strengths of this analysis include the large dataset spanning a clinically meaningful five-year surveillance window, use of validated disproportionality metrics (ROR and PRR) with pre-specified signal thresholds, comprehensive coverage of cardiac MedDRA preferred terms, and transparent reporting of null findings (non-significant signals) alongside positive results.

Limitations are inherent to the FAERS methodology. Spontaneous reporting is subject to underreporting (estimated completeness 1-10% for most AEs), notoriety bias (stimulated reporting following media attention), and variable report quality [22]. Exposure denominators are unavailable, precluding the calculation of incidence rates. Furthermore, structured causality assessment data (e.g., Naranjo scale) is unavailable in the FAERS database; therefore, direct causality cannot be definitively established, and confounding by indication, comorbidity, and concomitant medications cannot be controlled. The two-drug comparator framework is appropriate for head-to-head signal analysis but does not account for background population differences. Tirzepatide's much higher total reporting volume, partly reflecting its market entry during a period of intense GLP-1 media attention, may introduce reporting dynamics not present for semaglutide. Finally, while MedDRA preferred term mapping was applied systematically, some degree of miscoding in original reports is unavoidable.

## Conclusions

This pharmacovigilance disproportionality analysis of FAERS data from 2022 to 2026 identifies statistically significant semaglutide-disproportionate cardiovascular AE signals across eight of 12 MedDRA cardiac preferred terms, including the overall cardiac AE category and fatal outcome. The strongest individual signals were observed for congestive heart failure, cardiac failure, cardiac arrest, and atrial fibrillation.

Critically, these signals are best interpreted as markers of differential patient population characteristics, particularly the preferential prescribing of semaglutide to older patients with established cardiovascular disease, rather than evidence of pharmacotoxic cardiovascular harm. They do not contradict the cardiovascular mortality benefit demonstrated in the SELECT trial. Tirzepatide's cardiac AE profile is characterized by a proportionally lower but non-trivial burden of palpitations and tachycardia, potentially reflecting GIP receptor-mediated chronotropic effects.

These findings underscore the critical importance of cardiovascular monitoring for both agents in real-world clinical practice, the need for exposure-adjusted pharmacovigilance methods as prescription volume data become available, and the urgency of tirzepatide cardiovascular outcomes trial data. Head-to-head cardiovascular outcomes trials directly comparing these agents in indication-matched populations would provide the definitive evidence needed to resolve residual uncertainty in this rapidly evolving therapeutic landscape.
